# Psychometric Properties of the Generalized Anxiety Disorder Questionnaire - IV (GAD-Q-IV) in Postpartum Mothers

**DOI:** 10.1037/pas0000443

**Published:** 2017-02-20

**Authors:** Mark E. Pierson, Jason M. Prenoveau, Michelle G. Craske, Elena Netsi, Alan Stein

**Affiliations:** 1 Center for Social Organization of Schools, School of Education, Johns Hopkins University; 2 Department of Psychology, Loyola University Maryland; 3 Anxiety Disorders Research Center of UCLA, University of California, Los Angeles; 4 Department of Psychiatry, University of Oxford

**Keywords:** generalized anxiety disorder, postpartum anxiety, factor analysis, screening measure

## Abstract

Generalized anxiety disorder (GAD) is a mental disorder of which the main feature is persistent and impairing worry. GAD symptoms are common for women during the postpartum period and GAD prevalence rates have been reported as higher in postpartum mothers than in the general population. Currently, little psychometric evidence exists for a screening measure to detect the possible presence of diagnosable GAD for postpartum women. The purpose of this investigation was to gather psychometric information for the Generalized Anxiety Disorder Questionnaire–IV (GAD-Q-IV; Newman et al., 2002) with a sample of postpartum mothers. Factor analyses were conducted to determine the factor structure of the GAD-Q-IV in postpartum women. Receiver-operating characteristic (ROC) analysis was used to determine a range of potential GAD-Q-IV cut-off scores for detecting the likely presence of GAD in postpartum women. Results from this study provided evidence to justify a 1-factor structure for the GAD-Q-IV responses from postpartum women, which demonstrated structural, metric, and scalar invariance over time. Findings from these analyses provided evidence of incremental validity, as there was a significant increase in predicting GAD diagnoses when GAD-Q-IV responses were used compared with another measure of postpartum depression. Last, using ROC analysis, a range of GAD-Q-IV cut-off scores was determined, which can be applied to screening for the likely presence or absence of GAD in postpartum women. The evidence presented in this study suggests that the GAD-Q-IV could be a viable screening measure used to identify the likely presence of GAD in postpartum women so that further evaluations and treatments can be recommended.

## Public Significance Statement

The present investigation suggests that postpartum women experience higher levels of anxiety than the general population, which has a negative impact on their lives as well as their newborn children. We have provided evidence that physicians can use a measure of generalized anxiety disorder (GAD) to detect the likely presence of the disorder in postpartum women so that treatments can be recommended.

Generalized anxiety disorder (GAD) is a mental disorder with a main feature of excessive, impairing worry associated with three (or more) of the following six symptoms: restlessness or feeling keyed up or on edge, being easily fatigued, difficulty concentrating or mind going blank, irritability, muscle tension, and sleep disturbance (American Psychiatric Association [APA], 2013). Beyond the negative impact of pathological worry, many individuals with GAD experience physiological symptoms, such as an exaggerated startle response and somatic symptoms (e.g., sweating, nausea, and diarrhea; APA, 2013). GAD symptoms are especially common for women during the postpartum period (Phillips, Sharpe, Matthey, & Charles, 2009) and Wenzel, Haugen, Jackson, and Brendle (2005) found 1-year GAD prevalence of 8.2% for postpartum mothers in their sample (19.7% had subsyndromal GAD), whereas the 1-year GAD prevalence rate in the general population has been estimated to be between 1.6–3.0% (Craighead, Miklowitz, & Craighead, 2008). GAD is the most prevalent anxiety disorder for mothers during the first year after childbirth (Wenzel, 2011), as mothers experience excessive worry about their financial needs, physical appearance, domestic duties, sexual adjustment (Wenzel et al., 2005), how they will be able to care for their child (e.g., breastfeeding and soothing; Wenzel, 2011), and their newborn child’s well-being (Andrews et al., 2010).

In addition to the negative effects that GAD has on mothers suffering from this disorder, maternal GAD is associated with deficits in childhood development. Postpartum mothers with GAD are less responsive to their newborns (Stein et al., 2012) and mothers with maternal anxiety express less affection toward their children (Grant et al., 2012). Postpartum anxiety (i.e., not necessarily GAD, but anxiety symptoms in general) can disrupt mother–child bonding and attachment formation (Dawson, Ashman, & Carver, 2000). Anxiety disorders are likely to be transmitted from mother to child (Martini, Knappe, Beesdo-Baum, Lieb, & Wittchen, 2010) and the likelihood for long-term neurodevelopmental deficits for the children of mothers suffering from anxiety disorder are increased (Glover, Bergman, & O’Connor, 2008). When postpartum disorders are untreated in mothers, their children are at high risk for psychiatric, behavioral, and academic problems (Chase-Brand, 2008).

Mood disorders (e.g., major depression and generalized anxiety) are among the most underidentified, underreported, and untreated postpartum diagnoses (Goodman & Tyer-Viola, 2010). In North America, most women consider their obstetricians and/or gynecologists (OB/GYNs) their primary health-care provider(s) during pregnancy and postpartum visits (Hanusa, Scholle, Haskett, Spadaro, & Wisner, 2008). Though the role for OB/GYNs detecting psychiatric disorders in postpartum women is increasing (Weissman et al., 2004), many symptoms associated with pregnancy overlap with mood-disorder symptoms (e.g., somatic complaints, fatigue, and appetite changes), which makes accurately identifying disorders in postpartum women difficult (Lusskin, Pundiak, & Habib, 2007). Therefore, it is imperative to have an instrument that is able to aid in the identification of those who are likely to present with GAD postpartum. Richardson and Puskar (2012) suggested that the use of brief screening tools might enhance earlier identification of mental health diagnoses in postpartum women.

Postpartum GAD has been considerably less studied than postpartum depression (PPD; Hanusa et al., 2008). Like GAD, PPD has negative effects for mothers and their children (O’Hara, 2009). However, a wide-scale screening movement has shown to be pragmatic in the detection (Zubaran et al., 2010) and treatment (Delatte, Cao, Meltzer-Brody, & Menard, 2009) of PPD. Just as a screening paradigm has been used for PPD, an exigent need exists to expand this model to screen for postpartum GAD. To do so, a validated screening questionnaire that encompasses all of the symptomology of GAD is needed. If GAD is identified and treated during the postpartum period, some of the chronic (Prenoveau et al., 2013) and deleterious effects of GAD (Boschen, 2011) and negative childhood outcomes (Glasheen, Richardson, & Fabio, 2010) might be prevented. When health-care professionals appropriately diagnose GAD, adequate treatments can be recommended (Van der Heiden, Methorst, Muris, & Van der Molen, 2011).

To effectively screen for GAD, health professionals need an instrument that provides valid and reliable scores for anxiety among postpartum women (O’Hara et al., 2012). Though more research has been conducted with respect to postpartum GAD in recent years, little research has been conducted to evaluate a screening questionnaire to detect the likely presence of GAD postpartum. Meades and Ayers (2011) have recommended a method for addressing this limitation: use of an instrument developed with another population and collecting psychometric information with postpartum mothers. Simpson, Glazer, Michalski, Steiner, and Frey (2014) applied this method and investigated the psychometric properties of the GAD 7-Item Scale (GAD-7; Spitzer, Kroenke, Williams, & Löwe, 2006) using responses from 240 pregnant (*n* = 155) and postpartum (*n* = 85) women. Although Simpson et al. found that the GAD-7 was better than the Edinburgh Postnatal Depression Scale (EPDS; Cox, Holden, & Sagovsky, 1987) at differentiating between those with and without GAD in their sample, the GAD-7’s performance was typically in the “fair” range as opposed to “good” or “excellent.” They were able to detect the likely presence of GAD within their sample from scores on the GAD-7, with 61.3% sensitivity and 72.2% specificity, and having an optimal cut-off score of 13. Thus, the GAD-7 did not identify nearly 40% of the pregnant and postpartum women whom experienced psychiatrists had diagnosed with GAD. This is substantially more than the 11% missed by the GAD-7 in a sample of adults recruited from primary care (Spitzer et al., 2006). Furthermore, Simpson et al. (2014) found the area under the curve (AUC) to be 0.71 and 0.74 for detecting GAD alone and GAD with comorbid major depressive disorder (MDD), values which were just above the borderline between low and moderate accuracy (0.70). Although the GAD-7 is an easily administered tool, in part because of how few items it has, it is possible that its ability to accurately identify those with GAD in pregnant and postpartum women is degraded because it does not assess all the features of syndromal GAD.

The Generalized Anxiety Disorder Questionnaire-IV (GAD-Q-IV; Newman et al., 2002) is a nine-item self-report instrument that encompasses the entire clinical syndrome of GAD (Rodebaugh, Holaway, & Heimberg, 2008). The original Generalized Anxiety Disorder Questionnaire (GAD-Q; Roemer, Borkovec, Posa, & Borkovec, 1995) was developed based on the GAD criteria from the *DSM–III–R* (APA, 1987). Slight changes in the GAD criteria between the *DSM–III–R* and the *DSM–IV* (APA, 1994) lead to the revision and creation of the GAD-Q-IV (Newman et al., 2002). For example, the *DSM–III–R* dictated that pathological worry needed to be unrealistic or excessive, individuals needed to worry about two or more life circumstances, and it allowed for the presence of six of 18 possible symptoms (e.g., sweating or cold clammy hands). On the other hand, the *DSM–IV* (as well as the *DSM–IV–TR*; APA, 2000) and *DSM–5* (APA, 2013) require pathological worry to be excessive and uncontrollable, individuals must worry about a variety of events or activities, and individuals need to exhibit at least three out of six primary symptoms (Note: All of the six primary symptoms were taken directly from the *DSM–III–R* GAD criteria). Newman et al. (2002) used the original items from the GAD-Q and made adjustments to the items based on the shift of GAD criteria from the *DSM–III–R* to the *DSM–IV* to create the GAD-Q-IV. Though the GAD-Q-IV was developed for *DSM–IV–TR* GAD criteria, the *DSM–5* criteria for GAD remain virtually identical (Parry, 2013).

Newman et al. (2002) compared GAD-Q-IV scores with a clinical interview (ADIS-IV; Grisham, Brown, & Campbell, 2004) in a sample of college students (*N* = 143). Comparing self-report measure responses (in this case, using the GAD-Q-IV; Newman et al., 2002) to clinical interview diagnoses is the “gold-standard” validation method for self-report measures (Meades & Ayers, 2011; Rodebaugh et al., 2008). The comparison of the screening-measure scores and clinical interview results yielded a recommended cut-off score of 5.7 for the GAD-Q-IV, which yielded a sensitivity of 83% and a specificity of 89%, meaning that 11% of the college-student responses were incorrectly categorized as having GAD when they did not have the diagnosis (i.e., a false positive; Newman et al., 2002). One critique of this cut-off score is that it is possible for an individual to score above a 5.7, yet not endorse all of the diagnostic criteria of GAD. Therefore, caution may be warranted.

Data collected from college students using the GAD-Q-IV (Newman et al., 2002) have provided evidence of internal consistency (Cronbach’s α = .83; Rodebaugh et al., 2008), test–retest reliability (κ = .64 over 2 weeks; Newman et al., 2002), convergent validity (*r* = .66) with the Penn State Worry Questionnaire (PSWQ; Meyer, Miller, Metzger, & Borkovec, 1990), and divergent validity (*r* = .26) with the Zung Self-Rating Depression Scale (SDS; Zung, 1965). Rodebaugh et al. (2008) found that responses from a sample of college students (*N* = 1,038) to the GAD-Q-IV are best represented by a one-factor structure, which accounted for 25% of the variance of GAD-Q-IV item responses. Robinson, Klenck, and Norton (2010) found this one-factor structure of the GAD-Q-IV to be consistent among various racial/ethnic groups for another sample of college students (*n* = 585), which suggests that the GAD-Q-IV measures the construct of GAD uniformly across ethnic groups. The factor structure for the GAD-Q-IV is unknown for postpartum women, hence exploratory analyses were necessary.

If adequate psychometric properties are found for the GAD-Q-IV (Newman et al., 2002) in postpartum mothers (i.e., in the present and future studies), health-care professionals could use the GAD-Q-IV as a diagnostic screening instrument to detect the likely presence of GAD in postpartum mothers and recommend treatment(s) to prevent the chronic and detrimental effects of GAD in this population. The purpose of this investigation was to estimate internal consistency reliability, determine and confirm the factor structure, test for measurement invariance over time, and provide evidence of incremental validity for GAD-Q-IV responses provided by our sample of women postpartum.

## Method

### Sample and Participant Selection

Data were collected from mothers recruited from postdelivery wards at a large teaching hospital and surrounding health centers in England. Participation eligibility included mothers who were 18 years or older, spoke sufficient English, lived within 35 miles of the recruiting hospital, planned to be the infant’s principal caretaker, and had delivery of an infant after ≥35 weeks gestation, baby weighed ≥2,000 g at birth, and had no life-threatening medical complications. Written informed consent was collected from all participants after the institution’s ethics committee approved the current study for data collection.

At 9 weeks postpartum (9WP), 2,233^1^ mothers were administered two screening questionnaires: the EPDS (Cox et al., 1987) and the GAD-Q-IV (Newman et al., 2002, both described below) to identify those who likely had MDD, GAD, or both. To ensure there were adequate numbers of participants with and without these disorders for the larger project this study is part of, mothers who scored high on either screening questionnaire (>12 on the EPDS, >5.7 on the GAD-Q-IV; cut-off scores suggested by the original authors cited below), and a randomly selected group of women who scored below the cut-off on both questionnaires, were selected for diagnostic interviews using the Structured Clinical Interview for the *DSM–IV–TR* (SCID-I-RV; First & Gibbon, 2004, described below) at 3 months postpartum (3MP). Four clinical interviewers were responsible for administering the SCID-I-RV to our sample of postpartum women. At 3MP, a total of 296 participants were recruited into the larger study; based on SCID-I-RV interviews, 41 were diagnosed with both MDD and GAD, 80 with GAD without MDD, 40 with MDD without GAD, and 135 did not meet criteria for any current or past disorders. At 6 months postpartum (6MP), mothers were again administered the GAD-Q-IV and EPDS and were reinterviewed using the SCID-I-RV, although 42 participants within our sample did not return for the follow-up assessments and clinical interview at that time.

The majority of the sample that was recruited into the larger study at 3MP gave birth for the first time (i.e., primipara; 60.81%, *n* = 180), was Caucasian (91.89%, *n* = 272), reported their primary language as English (90.88%, *n* = 269), and reported a father in the household (80.07%, *n* = 237). The mean age was 31.55 years (*SD* = 5.06). A large proportion of our sample held an undergraduate degree (44.26%, *n* = 131), a postgraduate degree (15.88%, *n* = 47), or fewer educational or academic qualifications. The majority of our sample of postpartum mothers either worked in managerial or professional occupations (54.39%, *n* = 161) or intermediate occupations (19.59%, *n* = 58), and the remainder of our sample either worked in routine, manual occupations or were unemployed students.

### Measures

#### The GAD-Q-IV

The GAD-Q-IV (Newman et al., 2002), which is a nine-item self-report questionnaire, was used to assess GAD symptoms. The GAD-Q-IV total score is calculated by adding responses to five *yes* (1) and *no* (0) items (e.g., “Do you experience excessive worry?”), adding 1/3rd point for each of the possible six open-ended worry topics the participant may have listed (up to 2 points), 1/3rd point for each GAD *DSM* symptom endorsed (up to 2 points), and adding the responses to two Likert-type items. One of the yes/no items included whether the participant had been bothered by excessive worry for the past 3 months. Possible GAD-Q-IV scores range from 0 to 12, with higher scores representing greater GAD symptom severity. Data collected from college students with the GAD-Q-IV have provided evidence of internal consistency (Cronbach’s α = .83), test–retest reliability (κ = .64 over 2 weeks; Newman et al., 2002), and convergent and divergent validity (Rodebaugh et al., 2008).

#### The EPDS

The EPDS (Cox et al., 1987) is a 10-statement (e.g., “I have been so unhappy that I have been crying”) self-report measure with Likert-type responses ranging from 0 (*no, never*) to 3 (*yes, most of the time*) was used to assess severity of depressive symptoms. It is worth noting that some items, depending on the content of the statement or responses, needed be reverse-coded to appropriately interpret the results (see Cox et al., 1987 for further detail). Total scores are derived by adding all 10 responses and range from 0 to 30, with higher scores representing greater severity of depressive symptoms. Scores from the EPDS have provided evidence of split-half reliability (*r* = .88), internal consistency (Cronbach’s α = .87; Cox et al., 1987), and convergent and concurrent validity (Boyd, Le, & Somberg, 2005).

#### The SCID-I-RV

The SCID-I-RV (research version; First & Gibbon, 2004) is a structured interview used for diagnosing lifetime and current psychiatric disorders based on *DSM–IV–TR* (APA, 2000) criteria. The SCID-I-RV was used in the present study to assess the presence or absence of GAD and MDD. Diagnoses from the SCID-I-RV have demonstrated good interrater reliability for both MDD (κ = .66) and GAD (κ = .75; Lobbestael, Leurgans, & Arntz, 2011).

### Data Analysis

Exploratory and confirmatory factor analyses (EFA and CFA, respectively) were conducted with Mplus (Version 5.0; Muthén & Muthén, 2007). Randomization was used to divide participants into two subsamples from the 9WP data set: Subsample 1 (*n* = 1,117) was used for EFA and Subsample 2 (*n* = 1,116) was used for CFA. Nearly half of the overall sample that was recruited (*N* = 2,233) reported a father in the household (46.31%, *n* = 1,034) and the mean age of the mothers was 32.62 years (*SD* = 5.28). Parallel analysis was used to determine the number of factors to be extracted because it is considered one of the most accurate factor-retention methods (Hayton, Allen, & Scarpello, 2004) and functions well for categorical indicators (Garrido, Abad, & Ponsoda, 2016). The factor structure extracted from Subsample-1 data was used to guide which items would load onto which factors. A factor loading above .30 was the criterion to assign GAD-Q-IV (Newman et al., 2002) items to a factor. The factor structure found from EFA was cross-validated with the GAD-Q-IV responses from Subsample 2 using CFA. CFA was used to examine measurement and metric invariance with time (from 9WP to 6MP) of the factor structure. To test for incremental validity, logistic regression was used to determine whether GAD-Q-IV responses predicted GAD diagnoses above and beyond that of EPDS (Cox et al., 1987) responses with the 6MP data (*n* = 252).

Polychoric correlations and the robust weighted least-squares estimator (WLSMV) was used for both EFA and CFA as is recommended for analyzing measures with both categorical and continuous items (Brown, 2006). GAD-Q-IV (Newman et al., 2002) Likert-type items were treated as continuous and the remainder of the items were treated as categorical for all EFA and CFA analyses. Missing data were accommodated in both EFA and CFA models using full-information maximum likelihood under the assumption of missing at random (Muthén & Muthén, 2011). The Tucker–Lewis incremental fit index (TLI; Tucker & Lewis, 1973), comparative fit index (CFI; Bentler, 1990), and the root mean-square error of approximation (RMSEA; Steiger, Shapiro, & Browne, 1985) were used to infer model fit for all CFA analyses. TLI and CFI values approaching one (i.e., TLI and CFI ≥ .95) imply good model fit (Hu & Bentler, 1999), whereas RMSEA values approaching zero (i.e., RMSEA ≤ .05) indicate good model fit (Browne & Cudeck, 1992). Last, the 6MP dataset was used to examine the sensitivity and specificity of the GAD-Q-IV for predicting the presence or absence of GAD with receiver-operating characteristic (ROC) analyses using SPSS (version 22).

## Results

See Table 1 for means, standard deviations, Cronbach’s αs, and intercorrelations for the GAD-Q-IV and EPDS responses (Cox et al., 1987) at 9WP and 6MP.[Table-anchor tbl1]

### GAD-Q-IV Factor Structure in Subsample 1

An EFA was conducted on the GAD-Q-IV (Newman et al., 2002) responses with postpartum women using Subsample 1 (*n* = 1,117) at 9WP. To conduct parallel analysis (Hayton et al., 2004), eigenvalues were extracted from randomly generated data; the first two eigenvalues were 1.14, 95% CI [1.09, 1.19] and 1.10, 95% CI [1.06, 1.13]. The first eigenvalue from the Subsample-1 EFA (5.78) was greater than would be expected by chance, given the confidence interval of the first eigenvalue of the randomly generated data. The second eigenvalue (0.81) was not greater than would be expected by chance, given the confidence interval of the second eigenvalue of the randomly generated data. Therefore, only one factor was retained to represent the covariation among responses to GAD-Q-IV Items. The one underlying latent factor, which we call GAD Symptoms, accounted for 60.79% of the variance in responses to the nine GAD-Q-IV items.

### CFA of One-Factor GAD-Q-IV Model in 9WP Subsample 2

Results from Subsample-1 EFA were used to specify the one-factor model for Subsample 2 (*n* = 1,116). All nine items of the GAD-Q-IV (Newman et al., 2002) were specified to load onto one latent factor. Two cases were excluded from this analysis as a result of missing data from all nine GAD-Q-IV items. The one-factor CFA model for Subsample 2 was an adequate fit to the data for two of the fit indices, but was not an adequate fit based on the RMSEA, χ^2^(17) = 229.48, *p* < .001, RMSEA = .106, CFI = .94, TLI = .97.

Modification indices revealed evidence of correlated residuals between Item 8 (“How much do worry and physical symptoms interfere with your life, work, social activities, family, etc.?”) and Item 9 (“How much are you bothered by worry and physical symptoms?”); modification index = 59.90. It is likely that the covariance of these items not accounted for by the latent factor was due to method effects, possibly stemming from shared content, the shared response set (these two items shared a Likert-type response scale that is different from the other items), or some combination of both. Modification indices also revealed evidence of correlated residuals between Items 1 and 2 (modification index = 76.83), 4 and 5 (modification index = 46.26), 6 and 7 (modification index = 20.59), and 7 and 8 (modification index = 14.70). Closer examination revealed that residual covariance for each of these item pairs was also likely due to method effects as a result of overlapping content. For example, both Items 1 and 2 ask about excessive worry. Thus, the model was modified to allow the covariances between each of these item pairs to be freely estimated. These modifications resulted in a significant improvement in fit, Δχ^2^(4) = 164.14, *p* < .001, and the resulting model fit the data well, χ^2^(16) = 51.07, *p* < .001, RMSEA = .044, CFI = 0.99, TLI = 1.00.

To ensure that these residual covariances were not the result of chance characteristics of the data in Subsample 2, they were tested in Subsample 1. Similar to Subsample 2, the one-factor CFA model (without allowing the covariances between each of the five item pairs to be freely estimated) was an adequate fit to Subsample-1 data for two of the fit indices, but a poor fit based on the RMSEA, χ^2^(19) = 213.64, *p* < .001, RMSEA = .096, CFI = 0.93, TLI = 0.97. Modifying the model to allow the covariances between each of the five item pairs identified in Subsample 2 to be freely estimated resulted in a significant improvement in fit, Δχ^2^(4) = 151.60, *p* < .001, and the resulting model fit the data well, χ^2^(17) = 57.34, *p* < .001, RMSEA = .046, CFI = 0.99, TLI = 0.99. Thus, these modifications were retained when we examined measurement invariance.

### Measurement Invariance of the GAD-Q-IV One-Factor Model With Time

The sample of 296 participants recruited into the larger project were used for invariance analyses, as they were the participants who were invited to return for 6MP assessments; 42 of these participants did not complete the GAD-Q-IV (Newman et al., 2002) at 6MP. Before examining configural invariance with time using multiple-groups CFA, the one-factor model established above (including correlated residuals to address method effects) was examined separately in both 9WP (*n* = 291 because five participants did not complete any of the GAD-Q-IV items) and 6MP (*n* = 254) samples. The model was an excellent fit to the data at both 9WP, χ^2^(13) = 15.40, *p* = .283 RMSEA = .025, CFI = 1.00, TLI = 1.00, and 6MP, χ^2^(15) = 12.76, *p* = .621 RMSEA = .000, CFI = 1.00, TLI = 1.00.

Multiple-groups CFA, with participant data at 9WP constituting one group and participant data at 6MP constituting the second group, was used to examine invariance of the one-factor model with time. Using multiple-groups CFA, the one-factor model was applied to both 9WP and 6MP data simultaneously. As recommended by Muthén (2006) for models with categorical variables, configural invariance was examined by freeing thresholds and factor loadings across groups (9WP comprised one group, and 6MP the other, for temporal invariance), setting the latent-factor mean to 0 in both groups, and fixing scale factors to 1 in both groups. This multiple-groups, one-factor GAD-Q-IV model fit the data well, χ^2^(14) = 11.87, *p* = .617, RMSEA = .000, CFI = 1.00, TLI = 1.00, providing evidence of configural invariance. Next, metric and scalar invariance were tested together as recommended by Muthén. To do this, factor loadings, thresholds, and intercepts were constrained to equality across time points, the latent-factor mean was fixed at 0 for 9WP and freed for 6MP, and the scale factors were fixed to 1 for 9WP and freed for 6MP. Applying these constraints did not result in a significant decrement in model fit, Δχ^2^(7) = 8.44, *p* = .30 and the metric and scalar invariant model was an excellent fit to the data, χ^2^(29) = 30.08, *p* = .410, RMSEA = .012, CFI = 1.00, TLI = 1.00. Given that there was no decrement in model fit between the baseline model and the model with constrained factor loadings, the one-factor GAD-Q-IV model displays metric and scalar invariance with time for postpartum women. See Table 2 for the standardized parameter estimates for the one-factor, metric, and scalar invariant model.[Table-anchor tbl2]

### Incremental Validity Between the GAD-Q-IV and the EPDS

To test for incremental validity, hierarchical logistic regression was used to determine whether GAD-Q-IV (Newman et al., 2002) responses predicted GAD diagnoses above and beyond that of EPDS responses (Cox et al., 1987) with the 6MP data (*n* = 252). All three diagnostic groups were included in this analysis (i.e., including controls). EPDS responses significantly predicted GAD diagnoses, Cox & Snell *R*^2^ = .19, χ^2^(1) = 51.45, *p* < .001. When GAD-Q-IV responses were subsequently entered into the model, there was a significant increase in the prediction of GAD diagnoses, Cox & Snell Δ*R*^2^ = .11, Δχ^2^(1) = 35.19, *p* < .001, *OR* = 1.58, 95% CI [1.34, 1.86].

### ROC Analysis

The ROC analysis was conducted with 6MP data to compare continuous GAD-Q-IV (Newman et al., 2002) scores to dichotomous GAD diagnoses (GAD vs. no GAD) as diagnosed by the SCID-I-RV (First & Gibbon, 2004). All three diagnostic groups were included in this analysis (i.e., including controls). These results were then used to determine potential GAD-Q-IV cut-off scores with comparable percentages of sensitivity (true positives) and specificity (true negatives). We found the GAD-Q-IV cut-off score with balanced levels of sensitivity (81%) and specificity (80%) to be 6.38 for our sample of postpartum women. Please see Table 3 for additional ranges of GAD-Q-IV cut-off scores and their respective sensitivity and specificity values.[Table-anchor tbl3]

## Discussion

Currently, little evidence exists to show that responses from a measure can reliably and validly be used to screen for the presence of diagnosable GAD for postpartum women (Meades & Ayers, 2011). Our results provide sufficient evidence to justify a one-factor structure for GAD-Q-IV (Newman et al., 2002) responses from postpartum women and establish that GAD-Q-IV items are related to the underlying construct of GAD Symptoms in the same manner over time for postpartum women. The one-factor structure is consistent with previous research conducted with a sample of college students (Rodebaugh et al., 2008). Because only one construct is being measured, a total score—using Newman et al.’s (2002) scoring method—of the GAD-Q-IV responses could be used to assess GAD Symptoms for postpartum women.

We found GAD-Q-IV responses in postpartum women to possess configural, metric, and scalar invariance over time, from 9WP to 6MP. Given this evidence of measurement invariance with time, changes in GAD-Q-IV scores during the postpartum period likely represent actual changes in GAD symptomology for postpartum women and not changes in how the items function with time. Thus, researchers and health-care professionals can use the GAD-Q-IV to assess postpartum women over time and be confident that they are measuring the same underlying construct of GAD Symptoms. This would be especially important when monitoring symptoms as part of treatment for GAD.

Results from our hierarchical logistic regression analysis revealed that GAD-Q-IV (Newman et al., 2002) responses accounted for significantly more variation in GAD diagnoses than that of EPDS (Cox et al., 1987) response scores alone; the magnitude of this difference was quite large. These results provide evidence of incremental validly, such that GAD-Q-IV scores are more appropriate for assessing GAD in postpartum then EPDS scores. This is an important finding, given the fact that GAD and MDD symptoms are highly correlated (Cox et al., 1987; Simpson et al., 2014) and GAD and MDD are often comorbid (Goldberg, Kendler, Sirovatka, & Regier, 2010).

 Results from the ROC analysis indicated that the GAD-Q-IV scores were related to GAD diagnoses from structured clinical interviews. We found a GAD-Q-IV cut-off score with comparable levels of both sensitivity (81%) and specificity (80%) to be 6.38 for our sample of postpartum women. Thus, scores from the GAD-Q-IV seem to outperform those of the GAD-7 (Spitzer et al., 2006) in the identification of those postpartum women with, and without, GAD. Specifically, using the cut-off score with comparable levels of sensitivity and specificity, the present study found that approximately 19% of women with interview-assessed GAD were misdiagnosed as not having GAD. Just over twice this many women (38.7%) diagnosed with GAD by an experienced psychiatrist were not identified as having GAD by the GAD-7 (Simpson et al., 2014). Further, specificity was higher for GAD-Q-IV scores in the present study (80%) then those of GAD-7 scores in the past (72.2%; Simpson et al., 2014). Thus, GAD-Q-IV scores did a better job of identifying both those with GAD and those without GAD than GAD-7 scores.

The GAD-Q-IV cut-off score with balanced levels of sensitivity and specificity found within our sample of postpartum women was somewhat lower, but comparable to the 83% sensitivity and 89% specificity found with a sample of college students using the same measure (Newman et al., 2002). The sensitivity and specificity found in this study were also comparable to the 86% sensitivity and 78% specificity for identifying mothers with an MDD diagnosis using responses from the EPDS (Cox et al., 1987). Therefore, the GAD-Q-IV cut-off score with balanced sensitivity and specificity from our sample fit within the ranges of those found in other studies with the same measure, but a different population (Newman et al., 2002), and with the same population with a different construct being measured (Cox et al., 1987). Because the GAD-Q-IV has similar levels of sensitivity and specificity to a measure already being used for wide-scale screening for postpartum mothers (e.g., screening for PPD with the EPDS), this measure could be a viable screening instrument to assess the likely presence or absence of postpartum GAD. Further, it possesses better sensitivity and specificity in the prediction of GAD diagnoses than the only other measure of GAD symptoms that has been examined in a similar population (i.e., GAD-7; Simpson et al., 2014).

The GAD-Q-IV cut-off score with balanced sensitivity and specificity for postpartum women of 6.38 is higher than that of previous researchers’ cut-off score for college students of 5.7 (Newman et al., 2002). The cut-off score with comparable rates of sensitivity and specificity was likely higher for postpartum women because GAD symptoms, primarily excessive worry, are common among women during the postpartum period (Phillips et al., 2009), although these common worries may not be dysfunctional or impairing for some postpartum mothers. Hence, it is understandable that the threshold for meeting the criteria for diagnosable GAD—as measured by this GAD-Q-IV cut-off score—in postpartum women is higher than that of a college student population.

Although the GAD-Q-IV cut-off score in our sample was 6.38, we reported a range of cut-off scores so that health-care professionals can decide whether they are more interested in capturing a higher percentage of true positives. For example, the cut-off score of 5.04 elicited 93% sensitivity and 73% specificity for our sample of postpartum women. By lowering the GAD-Q-IV cut-off score to 5.04, health-care professionals can ensure that they are correctly identifying 93% percent of postpartum women who likely meet criteria for a GAD diagnosis. The tradeoff of selecting a lower GAD-Q-IV cut-off score is that the rate of false positives increases. Therefore, which cut-off score health-care professionals choose for detecting GAD in women postpartum depends on whether they are more concerned with capturing true positives or avoiding false positives.

### Clinical Implications

Postpartum GAD has been little studied compared with the larger body of literature examining PPD (Hanusa et al., 2008); however, a wide-scale screening movement has shown to be useful in the detection (Zubaran et al., 2010) and treatment (Delatte et al., 2009) of PPD. Just as the more common postpartum screening paradigm has proved to be pragmatic for the detection of PPD, so an exigent need exists to expand this model to screen for postpartum GAD, thereby increasing the chance of avoiding consequential deleterious effects (Amy Wenzel, 2011) for peri/postnatal mothers and their children (Dawson et al., 2000; Glasheen et al., 2010). The evidence presented in this study makes a strong case that the GAD-Q-IV (Newman et al., 2002) could be useful in assisting health-care professionals to screen for the possible presence of GAD in postpartum mothers. If postpartum women are screened for GAD with the GAD-Q-IV and surpass one of the cut-off scores recommended here, then health-care professionals can refer these women for further clinical evaluations (e.g., structured clinical interview) to determine whether a GAD diagnosis is warranted. If these women who undergo further clinical evaluation are then diagnosed with GAD, they can be referred to treatments needed to curtail their anxious symptoms and prevent further negative outcomes associated with this disorder. Health-care professionals can also use the GAD-Q-IV to detect the likely presence of GAD between 9WP and 6MP and be confident that they are measuring the same underlying construct of GAD symptoms over time, which may be helpful in determining whether treatments for anxiety are effective.

### Limitations and Future Directions

The sample obtained in the present study was limited by a lack of ethnic and cultural diversity. For example, 91.89% of the participants in our sample were Caucasian and all of our participants lived within 35 miles of the recruitment hospital in England. Because our sample was recruited from one geographical location, cultural diversity of postpartum women assessed within our study was limited and may not be a representative sample of all postpartum women. Future, cross-cultural research could be conducted to determine whether GAD symptomology varies over geographic space, between culturally diverse groups, or with available resources provided for women during the postpartum period. Furthermore, we did not include measures of other mental disorders that negatively impact women during the postpartum period, such as obsessive–compulsive disorder (OCD; Abramowitz, Schwartz, Moore, & Luenzmann, 2003; Wenzel, Gorman, O’Hara, & Stuart, 2001). Future researchers could assess OCD symptoms, as well, to determine whether these symptoms are similar or dissimilar to that of postpartum anxiety.

Another potential limitation is that the GAD-Q-IV (Newman et al., 2002) uses the *DSM–IV–TR* (APA, 2000) and *DSM–5* (APA, 2013) criterion of a 6-month duration of experienced excessive worry; however, we reduced this criterion to 3 months to measure GAD symptoms between time intervals. However, previous research has provided evidence that little difference exists between the required 6-month duration for GAD versus shorter durations (e.g., 3 months) in terms of age of onset, symptom severity or persistence, comorbidity with other mental disorders, or impairment in functioning (Lee et al., 2009). Finally, the GAD-Q-IV was not developed to assess GAD in postpartum mothers, but rather for the general population. This makes it difficult to determine how GAD-Q-IV items specifically relate to concerns for postpartum mothers (i.e., content validity of the GAD-Q-IV for this population). However, the fifth item of the GAD-Q-IV allows participants to list up to six open-ended topics about which they worry excessively or uncontrollably. Qualitative data from this item will likely assist health-care professionals to assess whether their patients’ excessive worries are more general or are related specifically to postpartum concerns.

### Conclusion

Results from our study provided sufficient evidence to justify a one-factor structure for GAD-Q-IV responses (Newman et al., 2002) from postpartum women. We found structural, metric, and scalar invariance for GAD-Q-IV responses over a 15-week period. Findings from the present study also provided evidence that scores from the GAD-Q-IV are better at identifying those with, and without, GAD than scores from the EPDS (Cox et al., 1987). Last, a range of GAD-Q-IV cut-off scores was determined that can be applied to screen for the likely presence or absence of GAD in postpartum women. In light of these findings, sufficient evidence has been provided demonstrating that the GAD-Q-IV has the screening capabilities needed to detect GAD in postpartum women. Screening postpartum women for GAD with the GAD-Q-IV will likely aid in the diagnostic process so that treatments can be recommended for these women and the deleterious consequences of GAD can be prevented for mothers and their families.

## Figures and Tables

**Table 1 tbl1:** Descriptive Statistics and Correlations for the GAD-Q-IV and EPDS at 9WP and 6MP

Time point	Measure	*N*	*M*	*SD*	α	1	2	3	4
1. 9WP	GAD-Q-IV	291	3.52	3.00	.82	—			
2. 9WP	EPDS	290	17.30	4.82	.87	***.74	—		
3. 6MP	GAD-Q-IV	254	4.53	3.78	.87	***.48	**.43	—	
4. 6MP	EPDS	254	18.14	5.84	.91	**.39	***.43	***.83	—
*Note*. 9WP = 9 weeks postpartum; 6MP = 6 months postpartum; GAD-Q-IV = Generalized Anxiety Disorder Questionnaire–IV; EPDS = Edinburgh Postnatal Depression Scale; α = Cronbach’s alpha. Listwise deletion was used to estimate internal consistency αs for both measures and time points. Variance and covariance matrices for item responses are available from the corresponding author upon request.									
** *p* < .01. *** *p* < .001.									

**Table 2 tbl2:** Standardized Parameter Estimates for a One-Factor, Scalar Invariant Model of the GAD-Q-IV

GAD-Q-IV item	Factor loading (λ)	*SE*	λ/*SE*	*p*
1. Excessive worry	.93	.03	37.33	.001
2. Worry intensity, frequency, etc.	.86	.04	23.55	.001
3. Difficulty controlling worry	.91	.03	32.18	.001
4. Excessive worry about minor matters	.77	.05	16.90	.001
5. List of frequent topics of excessive worry	.79	.03	27.39	.001
6. Worry more days than not (past 3 months)	.88	.03	28.55	.001
7. Bothered by GAD symptoms (past 3 months)	.75	.03	22.78	.001
8. Impairment of worry and physical symptoms	.78	.03	28.24	.001
9. Distress caused by worry and physical symptoms	.80	.03	31.11	.001
*Note*. GAD-Q-IV = Generalized Anxiety Disorder Questionnaire-IV; GAD = Generalized anxiety disorder. These factor loadings are based on a model with 9 weeks and 6 months postpartum data combined.				

**Table 3 tbl3:** Range of GAD-Q-IV Scores With Sensitivity and Specificity

Score	Sensitivity	Specificity
4.17	94.8%	70.1%
4.54	93.1%	70.6%
4.79	93.1%	71.1%
4.92	93.1%	71.6%
5.04	93.1%	72.7%
5.13	91.4%	73.2%
5.21	89.7%	73.7%
5.29	87.9%	73.7%
5.42	86.2%	74.7%
5.54	84.5%	74.7%
5.67	82.8%	75.3%
5.79	82.8%	75.8%
5.92	82.8%	77.3%
6.04	82.8%	77.8%
6.08	82.8%	78.9%
6.13	82.8%	79.4%
6.25	81.0%	79.4%
6.33	81.0%	79.9%
6.38	81.0%	80.4%
*Note*. 5.67 rounds to 5.7, which is the cut-off score recommended by Newman et al. (2002).		

## Footnotes

1 The sample consisted of the 2,202 subjects described in a previous study, plus an additional 31 subjects who served as pilots for the larger project. These 31 are included in the present analyses because they followed the same procedures as participants in the larger project for the data presented in the current study.
